# The Genetics of Coronary Artery Disease: A Vascular Perspective

**DOI:** 10.3390/cells12182232

**Published:** 2023-09-08

**Authors:** Leon N. K. Quaye, Catherine E. Dalzell, Panos Deloukas, Andrew J. P. Smith

**Affiliations:** William Harvey Research Institute, Faculty of Medicine and Dentistry, Queen Mary University of London, London EC1M 6BQ, UK; l.quaye@qmul.ac.uk (L.N.K.Q.); c.dalzell@smd20.qmul.ac.uk (C.E.D.); a.s.smith@qmul.ac.uk (A.J.P.S.)

**Keywords:** coronary artery disease, GWAS risk loci, vascular pathways, expression quantitative trait loci, massively parallel reporter assay, CRISPR-based gene-editing tools

## Abstract

Genome-wide association studies (GWAS) have identified a large number of genetic loci for coronary artery disease (CAD), with many located close to genes associated with traditional CAD risk pathways, such as lipid metabolism and inflammation. It is becoming evident with recent CAD GWAS meta-analyses that vascular pathways are also highly enriched and present an opportunity for novel therapeutics. This review examines GWAS-enriched vascular gene loci, the pathways involved and their potential role in CAD pathogenesis. The functionality of variants is explored from expression quantitative trait loci, massively parallel reporter assays and CRISPR-based gene-editing tools. We discuss how this research may lead to novel therapeutic tools to treat cardiovascular disorders.

## 1. Introduction

Despite decades of research into prevention and treatment, coronary artery disease (CAD) remains a leading cause of mortality worldwide [1]. A contributing reason for this is that we do not have a full understanding of the molecular pathways that lead to the development of atherosclerosis, the pathophysiological process underlying CAD. Atherosclerosis is a chronic inflammatory condition characterised by the accumulation of cholesterol-rich lipoproteins that form a plaque and compromise blood flow [2,3]. The development of atherosclerosis involves a complex interplay between environmental risk factors and genetic susceptibility, with heritability estimates for CAD of ~40–60% [4,5].

Human genetic studies have the potential to provide insight into disease-relevant cell types and their biological pathways that underpin the genetic contribution to CAD. Genome-wide association studies (GWAS) are a powerful technique to uncover genetic variants influencing risk for common human traits and diseases, implicating genes and pathways involved in pathogenesis [6]. However, a major bottleneck in translating genetic association findings into new therapeutics is the difficulty in defining the causal genes, often necessitating functional validation of many candidate genes located within the GWAS-identified loci. This step is further complicated by the vast majority of index GWAS variants falling within noncoding regions of the genome and their strong linkage disequilibrium (LD) with many other surrounding single-nucleotide polymorphisms (SNPs) making the identification of the causal variant a greater challenge. As a result, despite many significant associations, the underlying genes and related molecular mechanisms at most loci remain unknown [7]. A substantial number of GWAS-associated susceptibility loci encompass regulatory elements that are specific to disease-relevant cell types and states which emphasises the importance of identifying the appropriate causal cell types and the environment to which they are exposed in disease to fully appreciate the biological pathways responsible for the genetic basis of CAD [8].

GWASs have now identified over 350 genomic loci associated with CAD at the genome-wide level of significance (*p* < 5 × 10^−8^) [9,10,11,12]. The first CAD risk locus to be discovered in a GWAS was the 9p21 locus which continues to be the strongest and most replicated association signal [13,14]. Although virtually inexistent amongst the African American population, a significant association has been previously reported with a distinct haplotype at the locus [12,15]. Acknowledging the need of larger sample sizes to detect risk variants with weaker effect sizes and/or lower minor allele frequency (MAF < 5%), the CARDIoGRAM and C4D consortia were created to enable collaborative meta-analyses, thereby increasing power and discovering may more CAD risk loci [16,17,18,19,20,21,22]. The ability to perform such large-scale genetic studies has been aided by the formation of large biobanks including Biobank Japan, UK Biobank and the Million Veteran Program (MVP) in the USA [10,23,24]. The latest two key CAD GWAS meta-analyses included information from over 1.6 million participants, and collectively discovered over 300 significant risk loci, demonstrating the power of GWAS genomic analysis in uncovering molecular pathways underlying this disease (Supplementary Table S1) [11,12]. The CARDIoGRAMplusC4D Consortium’s meta-analysis involved 1,165,690 individuals of primarily European ancestry, with 181,522 cases of CAD [11] reporting a total of 279 risk loci at a genome-wide level of significance. Pathway analysis has highlighted the major biological mechanisms implicated by these CAD risk loci including well-established mechanisms in CAD pathogenesis such as lipid metabolism and extracellular matrix structure and function. However, other important genes were linked to vascular cell function, migration, and proliferation as well as pathways connected to cell cycle signalling and early developmental processes. The power of multi-ancestry analysis to improve the rate of discovery and enhance our understanding of the genetic underpinnings of CAD for better risk prediction and therapeutic advances is clear. This was reinforced by results from the multi-ethnic GWAS conducted using data from the MVP and other studies integrating White, Black, and Hispanic individuals and identifying 95 novel loci at the time including nine on the X-chromosome [12]. Once again, in addition to traditional CAD pathways, the gene-set enrichment analyses indicated a role for mechanisms underlying basic cellular processes such as cell cycle, replication, and growth. Interestingly, analysis also suggested a shared biology with oncogenesis by noting that several important epithelial–mesenchymal transition (EMT) genes with proven roles in cancer pathophysiology are also candidate CAD genes that could alter risk by controlling vascular smooth muscle cell (VSMC) transitions in the development of atherosclerosis.

**Figure 1:**
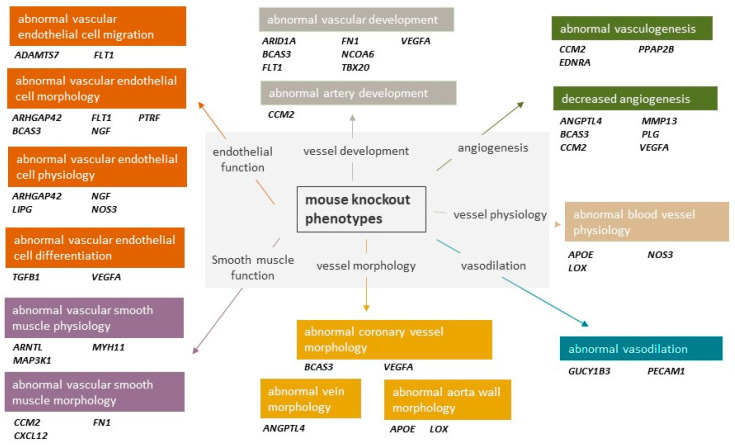
CAD GWAS genes implicated in vascular processes though knockout mouse models. Data adapted from Aragam et al. [11].

Two-thirds of the identified CAD risk loci were not associated with traditional risk factors such as circulating low-density lipoprotein (LDL) cholesterol levels or hypertension [25]. This implies that a substantial number of CAD susceptibility genes do not exert their effects through these well-established pathways, and undiscovered pathological mechanisms that are not targeted by current therapies may exist. Aside from more recognised pathways including inflammation, extracellular matrix remodelling and nitric oxide signalling, GWAS have uncovered the contribution of genes that regulate primary biological processes in vascular cells that form the blood vessel wall [11,20,21]. It is becoming increasingly evident that a significant part of the genetic risk for CAD is attributable to these vascular pathways that act directly through endothelial cells (ECs) and VSMCs cells which are key in the development and progression of atherosclerosis [26]. Studies looking at knockout mice for CAD GWAS genes have shown enrichment of vascular phenotypes including both endothelial and smooth muscle cell functions [11] (Figure 1).

This review will focus on CAD GWAS genes that have been implicated in vascular pathways, functional validation techniques and possibilities for emerging therapeutic avenues, with a focus on the more recently identified CAD loci.

### 1.1. Vascular CAD GWAS Genes and Pathways

**Figure 2:**
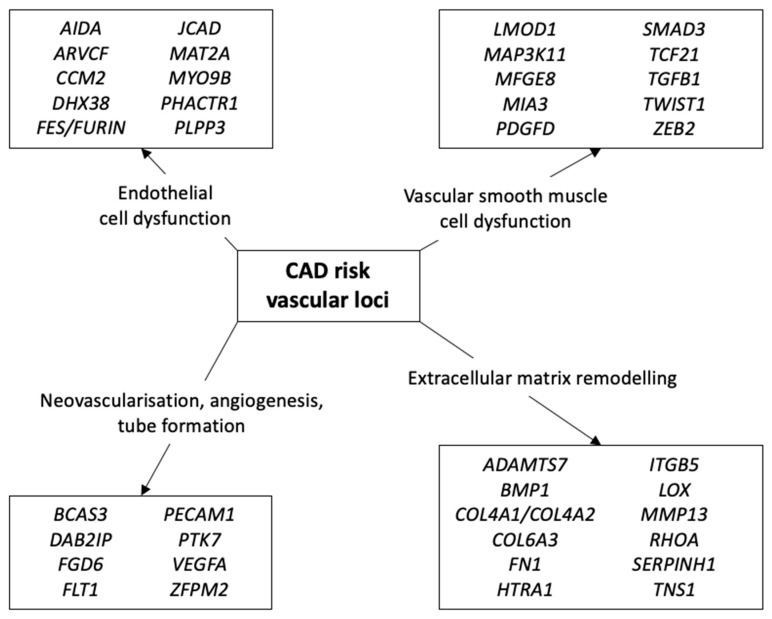
Genes from CAD GWAS studies are implicated in a number of vascular-related processes, including endothelial and smooth muscle cell dysfunction, neovascularisation, angiogenesis, tube formation, and extracellular matrix remodelling. Many of these genes are likely to be involved in several vascular processes.

Whilst many genes implicated in CAD pathogenesis from GWAS studies have no clear role based on current knowledge, several can be broadly categorised into four vascular-related processes: vascular endothelial cell dysfunction, vascular smooth muscle cell dysfunction, neovascularisation, and extracellular matrix remodelling, with many genes likely to have overlapping functions (Figure 2).

#### 1.1.1. Vascular Endothelial Cell Dysfunction

The innermost layer of blood vessels is formed of endothelial cells which play a key role in the pathophysiology of CAD [2]. Vascular endothelial cells provide a selective barrier preventing many macromolecules in the blood from entering the intima, control inflammatory responses, growth and migration of smooth muscle cells, as well as regulating vascular tone in response to haemodynamic changes [27]. Exposure to atherogenic stimuli including hyperlipidaemia, hyperglycaemia and a hypertensive environment initiates multiple functional pathological changes known as endothelial cell dysfunction, associated with atherosclerotic plaque development [28]. Activated endothelial cells result in intercellular permeability secondary to weakened cell–cell junctions, which in combination with increased production of adhesion molecules, allow monocyte attachment and migration into the intima. In addition to the recruitment of inflammatory cells, dysfunctional endothelial cells abnormally regulate the vasoactive molecules nitric oxide and endothelin-1 and exhibit other atheroprone characteristics such as impaired calcium signalling, increased production of reactive oxygen species and senescence [28,29].

Several CAD GWAS loci harbour genes that modulate endothelial cell functions providing support for the causal role of these pathways in disease risk [29,30,31,32].

##### JCAD

CAD risk variants located at the *JCAD* (junctional cadherin 5 associated, also known as KIAA1462) locus [33] were associated with increased gene expression in human arteries and *JCAD* knockout reduced the development of atherosclerosis in apolipoprotein E-deficient (ApoE^−/−^) mice [30]. *JCAD*, an endothelial cell–cell junctional protein, promoted endothelial dysfunction via YAP/TAZ activation, and this was proposed to drive the expression of inflammatory genes and facilitate monocyte adhesion to endothelial cells contributing to the formation of atherosclerotic plaques.

##### AIDA

An integrated map of gene expression, open chromatin regions and 3D interactions in human endothelial cells was created to assess the contribution of CAD-associated genetic variants that exert their effects via regulation of vascular endothelial functions [31]. This study provided evidence in favour of an atherogenic role at the *AIDA*/*MIA3* locus for the novel CAD candidate gene *AIDA* (Axin interaction partner and dorsalisation antagonist), hindering activation of the c-Jun N-terminal kinase (JNK) when overexpressed in zebrafish. The authors proposed that the CAD-associated risk variant at this locus acts via a TNFα-responsive regulatory element responsible for *AIDA* expression which in turn leads to endothelial cell dysfunction through its effects on JNK.

##### PLPP3

At the *PLPP3* (phospholipid phosphatase 3) locus, a CAD-associated regulatory variant was characterised influencing vascular endothelial phenotypes [17]. *PLPP3* is cardioprotective through its effect on endothelial function, including its conservation of monolayer integrity and inhibition of inflammation [34]. Haemodynamics play a crucial role in vascular homeostasis, and arterial regions prone to atherosclerosis display locally disturbed blood flow which activates endothelial cells [35]. The CAD-protective variant, located within an enhancer for *PLPP3*, upregulated enhancer activity under unidirectional flow by providing a binding site for the transcription factor Krüppel-like factor 2 (KLF2) [32]. This study demonstrated that human disease-associated genetic variants are capable of influencing key endothelial responses to blood flow.

##### MAT2A

CRISPR screens have recently been used to evaluate the effect of genomic regions at or near CAD-associated loci on vascular endothelial cell functions [29]. The study identified *MAT2A*, a methionine adenosyltransferase that catalyses the formation of S-adenosylmethionine from methionine and ATP, as a candidate CAD gene [36]. In TNFα-treated immortalised human aortic endothelial cells (teloHAEC), Cas9 targeting a synonymous variant in *MAT2A* enhanced ROS production, an atheroprone endothelial cell phenotype.

##### DHX38

This CRISPR screen also highlighted the possibility of endothelial cell senescence as a mechanism contributing to CAD. Deletion of *DHX38*, an RNA helicase involved in splicing, in endothelial cells, induced features of cellular senescence including restricted cell cycle progression, increased expression of *CDKN1A,* and enhanced β-galactosidase activity [29]. The authors did, however, acknowledge that despite their interesting results for the role of *DHX38* in contributing to CAD risk through endothelial cell dysfunction, the CAD association signal at this locus may be a result of variants in weak linkage disequilibrium that associate with LDL cholesterol.

##### ARVCF

A recent GWAS meta-analysis for CAD revealed 30 novel loci, including several linked to vascular pathways [11]. *ARVCF* (armadillo repeat protein deleted in velocardiofacial syndrome) belongs to the catenin family whose members have vital roles in the formation of adherens junction complexes that function to maintain cell–cell adhesion and tissue structure, whilst also allowing cell movement during tissue development or renewal. The presence of a nuclear localisation signal has led to suggestions that ARVCF could also behave as a signalling molecule moving between the plasma membrane and the nucleus [37,38]. The precise role of *ARVCF* in CAD is not yet clear; however, it might impact vascular disease risk through differential expression in endothelial cells and the corresponding effect on vascular wall biology [39].

##### MYO9B

Another CAD risk locus from this study led to the prioritisation of *MYO9B* (unconventional myosin-IXb) as a candidate gene, a myosin protein with a Rho-GTPase-activating function that has a role in cell migration [11,40]. CRISPR-Cas9 deletion was used to functionally validate the *MYO9B* locus in relation to CAD risk. The authors demonstrated the presence of a vascular tissue enhancer at the GWAS-associated locus which when deleted in immortalised human aortic endothelial cells resulted in a reduced expression of *MYO9B* and *HAUS8* and impaired wound healing. The CAD risk allele was linked to lower *MYO9B* expression, providing support for the proposed molecular mechanism linking this novel locus to CAD.

##### FES/FURIN

A CAD-associated locus that spans both *FES* and *FURIN* (*FES* Upstream Region) [20] has implicated these two genes in disease pathology, and although the mechanism is unclear, potential cells involved include endothelial cells, smooth muscle cells and monocytes. The CAD risk variant has been shown to modulate *FURIN* expression and affect monocyte–endothelial adhesion and migration [41], although chromatin signatures and eQTL analysis have implicated the involvement of *FES* in endothelial cells [42]. Genome-editing studies indicate a potential allele-specific interaction with inflammatory stimuli and *FES* expression in endothelial cells [29]. A study which used siRNA to knockdown *FES* showed increased migration in monocytes and VSMCs, whilst a Fes mouse knockout demonstrated increased size of atherosclerotic plaque including a higher content of monocyte/macrophages and SMCs [43].

#### 1.1.2. Vascular Smooth Muscle Cell Dysfunction

Vascular smooth muscle cells are a crucial component of blood vessel walls, enabling their contractile properties whilst also providing structural support. In non-disease states, vascular smooth muscle cells are quiescent and their primary function is the control of blood pressure, with their transcriptome reflecting genes required for contraction [44]. However, recent single-cell transcriptomic analyses have revealed widespread plasticity of cells within atherosclerotic plaques and there is accumulating evidence that smooth muscle cell state changes represent key molecular mechanisms underlying the pathophysiology of CAD [45,46,47]. In response to atherogenic stimuli and vascular stress, contractile smooth muscle cells are believed to switch to a dedifferentiated phenotype displaying increased migration, proliferation, and extracellular matrix synthesis [48].

Studies have begun to identify and characterise GWAS-identified CAD-associated genes involved in regulating these smooth muscle cell phenotypic transitions and have shed light on the importance of these pathways in atherosclerosis susceptibility [26,45,49,50,51,52]. According to the type of phenotypic change elicited, genetic variation effecting these processes can result in vascular smooth muscle cells having either disease-promoting or -limiting effects [51].

##### 9p21 Locus

The 9p21 locus, in proximity to *CDKN2B-AS1* (*ANRIL*), remains the strongest risk association for CAD, despite a lack of intermediate phenotypes to establish a clear mechanism. The closest protein-coding genes are *CDKN2A*/*B* and *MTAP*, which along with an interval downstream of *INFA21*, have been shown to physically interact with the enhancer-rich CAD risk locus in endothelial cells (HUVEC), suggesting long range gene regulation may be involved [53].

Despite potential functionality in endothelial cells, research also implicates VSMCs with a role in CAD pathogenesis, with an early study demonstrating differential expression of CDKN2A/CDKN2B/CDKN2B-AS1 in these cells [54]. Another study used induced pluripotent stem cells (iPSCs) to create VSMCs with homozygous risk and non-risk 9p21 haplotypes, examining the effect of deleting these haplotypes using genome editing [55]. It was found that deleting the risk haplotype restored the transcriptional profile of VSMCs to that resembling the non-risk haplotype. Along with genes involved in the cell cycle, DNA replication and repair, those involved with cell adhesion, muscle development and muscle contraction were differentially expressed, suggesting a potentially novel mechanism for CAD risk. Another study adopting a similar strategy to examine the effect of risk and non-risk haplotypes from iPSC-derived VSMCs demonstrated increased proliferation, migration and calcium phosphate deposits in cells harbouring the risk haplotype [56].

##### SMAD3 and TGFB1

The TGFβ signalling pathway has been implicated in CAD pathogenesis with risk alleles identified at *TGFB1*, *BMP1* and *SMAD3* loci [20]. TGFβ signalling plays an important role in vascular wall development [57] and VSMC differentiation [58], although debate remains whether TGFβ signalling is atheroprotective or atherogenic [59]. In vivo evidence suggests TGFβ is involved in VSMC proliferation through an SMAD3-dependent mechanism [60]. An in vitro study showed that SMAD3 controlled markers of differentiation in coronary VSMCs and proliferation [61]. The authors suggested the role of SMAD3 in pro-differentiation may result in disease plaque destabilisation with the possibility that another transcription factor, TCF21, a locus associated with CAD protection, may act in an opposing manner.

##### ZEB2

A recent study reported *ZEB2* as a novel CAD GWAS gene involved in phenotypic switching by identifying a smooth muscle long-distance enhancer within a CAD-associated GWAS signal [49]. *ZEB2*, a zinc finger homeodomain transcription factor, has a key role in another phenotypic switch, the epithelial–mesenchymal transition (EMT) observed in cancer and development, with several parallels to that observed in vascular smooth muscle cells [62]. It is also a binding partner for *SMAD3*, an established CAD-associated gene [63]. This study showed that Zeb2 is briefly expressed in mouse atherosclerotic smooth muscle cells as they de-differentiate and undergo transition to fibromyocytes followed by chondromyocytes. *ZEB2* exerts its effects on smooth muscle cell phenotype via chromatin remodelling that alters accessibility and interferes with Notch and TGFβ signalling. Smooth muscle cell-specific knockout of Zeb2 hindered the transition of smooth muscle cells into fibromyocytes but increased premature differentiation into chondromyocytes, a cell composition analogous to high-risk atherosclerotic lesions in human coronary arteries. This plaque vulnerability, through direct effects on the epigenome, may account for the higher risk of myocardial infarction found in individuals with polymorphisms associated with lower *ZEB2* expression in smooth muscle cells. The importance of pathways involved in vascular smooth muscle cell dysfunction and the role of EMT-regulating genes within this is further supported by the presence of multiple CAD GWAS signals close to other EMT-related genes, including *TGFB1*, *SNAI1* and *TWIST1* [49].

##### TWIST1

Transcriptomic profiling of genotyped human-derived vascular endothelial and smooth muscle cell pairs identified a link between a CAD risk locus and *TWIST1*. [50] *TWIST1* is a basic helix–loop–helix (bHLH) transcription factor involved in EMT and the development of coronary artery smooth muscle cells via upregulation of *TCF21*, another CAD-associated gene which also has a role in modulating smooth muscle cell phenotypes in diseased vessel walls [45,64]. The study demonstrated that disrupting the CAD-associated SNP reduced *TWIST1* expression and proposed that the minor (risk) allele generates an RBPJ binding site which, in combination with Notch signalling, promotes *TWIST1* transcription. This leads to smooth muscle cell phenotypic switching with increased cell proliferation and migration within the developing atherosclerotic lesion. Additionally, *TWIST1* has been associated with shear-stress-induced endothelial dysfunction, suggesting another potential role in CAD risk [65].

##### MIA3

The role of the *AIDA*/*MIA3* locus in CAD has been discussed in terms of endothelial dysfunction through *AIDA*; however, *MIA3* is also implicated in the modulation of vascular smooth muscle cell behaviour [51]. MIA3 is found at the endoplasmic reticulum exit site where it facilitates the secretion of molecules such as collagen from vascular smooth muscle cells [66]. It was shown that the CAD risk allele resulted in lower expression of *MIA3* and reduced proliferation of vascular smooth muscle cells in comparison to the non-risk allele [51]. MIA3 immunostaining in human coronary atherosclerotic lesions validated the authors’ hypothesis that reduced vascular smooth muscle cell proliferation results in the creation of a thin fibrous cap which is more prone to rupture, thereby carrying an increased risk of myocardial infarction.

##### TCF21

*TCF21*, an embryonic transcription factor, has been shown to have a protective function in the development of atherosclerosis through vascular smooth muscle cell phenotypic modulation [45]. Smooth muscle-specific knockout of *Tcf21* inhibited its phenotypic transition with reduced numbers of fibromyocytes in the atherosclerotic lesion and at the protective fibrous cap [45]. The authors reported a causal association between increased *TCF21* expression and reduced the risk of CAD and proposed that this protective influence is the result of migration of fibromyocytes into the atherosclerotic plaque and fibrous cap. A further study demonstrated that TCF21 modulates the smooth muscle cell phenotype by inhibiting the myocardin-serum response factor (MYOCD-SRF) pathway [67].

##### PDGFD

In contrast to the protective functions attributed to *ZEB2*, *MIA3* and *TCF21* genes, which act to ensure vascular smooth muscle cells transition to a fibromyocyte phenotype, *PDGFD* (platelet derived growth factor) is a GWAS-identified CAD-associated gene that is atherogenic [26,45,49,51]. A recent study demonstrated that *PDGFD* contributes to CAD risk by facilitating vascular smooth muscle cell expansion, migration and adoption of the chondromyocyte phenotype with calcification [26]. The authors established that the regulatory variant determined *PDGFD* expression through differential binding of the FOXC1/C2 transcription factor.

##### MFGE8 and MAP3K11

Two further GWAS-identified CAD-associated genes exerting their effects through smooth muscle cell behaviours are *MFGE8* (Milk Fat Globule-EGF factor 8), an integrin-binding glycoprotein, and *MAP3K11* (Mitogen-Activated Protein Kinase Kinase Kinase 11). The inhibition of *MAP3K11* resulted in reduced migration of vascular smooth muscle cells, a vital process in the development of atheromatous lesions [52]. The knockdown of *MFGE8* has also been shown to negatively impact the proliferation rate of vascular smooth muscle cells and those with genetic variation increasing *MFGE8* expression have a higher CAD risk [68].

#### 1.1.3. Neovascularisation

In addition to endothelial cell dysfunction and smooth muscle cell phenotypic switching, neovascularisation of the plaque is a crucial pathogenic event in atherogenesis as it is responsible for its growth and contributes to plaque instability, leading to thromboembolic consequences [69]. The inflammatory and relatively anoxic environment in atherosclerotic lesions stimulates the production of angiogenic factors that induce sprouting angiogenesis and encourage plaque progression and remodelling by ensuring adequate nutrients and oxygen to cells. However, these neocapillaries are fragile and prone to intraplaque haemorrhages that can destabilise and rupture plaques [70].

##### VEGFA and FLT1

VEGF-A (*VEGFA* gene), an endothelial-specific growth factor and potent angiogenic inducer, and the VEGF receptor 1 (*FLT1* gene) loci both associate with CAD [71]. VEGF-A has both beneficial and harmful roles in atherosclerosis. It stimulates expression of anti-apoptotic proteins and increases nitric oxide production as well as promoting re-endothelialisation at sites of injury thereby protecting endothelial cells and reducing regions that could trigger atherogenesis. Nonetheless, VEGF-A also promotes pro-atherogenic changes including increased endothelial permeability and expression of adhesion molecules resulting in monocyte adhesion, activation and transmigration into the blood vessel wall [72,73]. VEGF-A stimulates angiogenesis with associated haemorrhages and plaque instability. In animal models, VEGF-A accelerates atherosclerosis progression and anti-angiogenic agents have the reverse effect [69].

##### PTK7

A recently discovered CAD candidate gene from GWAS that is also linked to VEGF-A-induced angiogenesis is *PTK7* [11,74]. Protein tyrosine kinase 7 is a pseudokinase that is required for the activation and regulation of VEGFR-1 angiogenic signalling [74]. PTK7 forms a receptor complex with VEGFR-1 that has an essential role in endothelial cell migration and tube formation, both required for successful angiogenesis. Inhibition of *PTK7* expression by siRNA led to reduced VEGFR-1 phosphorylation and consequently impaired downstream signalling through Akt (AKT serine/threonine kinase) and FAK (focal adhesion kinase). Furthermore, overexpression of *PTK7* in endothelial cells in vitro triggered enhanced angiogenesis, whereas knockdown of *PTK7* by siRNA dramatically compromised VEGF-A-stimulated neovascularisation in vivo [74]. These results support the idea that PTK7 is a crucial element of the signalling pathway involved in VEGFR-1 mediated angiogenesis.

##### BCAS3

Another GWAS-identified CAD-associated gene involved in angiogenesis and vascular remodelling is *BCAS3* (Breast Carcinoma-Amplified Sequence 3) [19]. *BCAS3* encodes the Rudhira protein that controls cell polarity and migration of endothelial cells in angiogenesis through the activation of CDC42 and reorganisation of the actin cytoskeleton [75]. *BCAS3* mouse knockout models demonstrated severely compromised angiogenesis with abnormal expression of genes linked to key processes in angiogenesis including cell adhesion and invasion in addition to matrix organisation and degradation [76].

##### Extracellular Matrix Remodelling

Multiple steps are required for the formation of an atherosclerotic plaque and extracellular matrix (ECM) remodelling plays an integral role by expanding the intimal space and enabling the retention of LDL molecules [77,78].

##### FN1

GWASs have linked several extracellular matrix genes to CAD including *FN1* which encodes the glycoprotein fibronectin [71]. Fibronectin can be synthesised directly within the plaque or absorbed from the plasma and is involved in cell adhesion, migration, and proliferation [78]. Its role in atherosclerosis appears to be complex with actions in endothelial permeability and maintaining an inflammatory state as well as fibrous cap stability [79,80]. Mouse *FN1* knockouts in hepatic and haematopoietic cells demonstrate a reduced number of smaller, less lipid-rich plaques [80]. A recent study explored the underlying mechanism between a GWAS signal at the *FN1* gene and CAD risk [81]. The authors demonstrated that a SNP located within the *FN1* signal peptide sequence affected the secretion of the protein, showing that a coding variant linked to CAD can regulate function through post-transcriptional consequences. Higher plasma FN1 protein levels were also associated with reduced CAD risk suggesting a cardioprotective role.

##### MMP13

Another GWAS gene with a role in ECM remodelling that has been recently implicated in CAD pathogenesis is *MMP13* [11]. This encodes matrix metalloproteinase-13, an interstitial collagenase that affects intraplaque collagen content and organisation thereby altering the stability of atherosclerotic plaques and their susceptibility to rupture [11,82].

## 2. Characterisation of Functional Vascular CAD Variants

One of the key hurdles in assigning causal genes and pathways to GWAS signals is the identification of the functional variant which is hampered by the extent of LD in the locus. Indeed, the role of long-range interactions between enhancers and gene targets can often result in the nearest gene to the functional variant not being the target gene. Several methods have been used to try to pinpoint functional variants and to determine their functionality, and these will be discussed in context of CAD GWAS signals where the target may be working through a vascular pathway.

### 2.1. Functionally Informed Fine-Mapping Studies

Prior to experimental approaches, statistical approaches can refine identified associations by exploiting genomic annotations such as chromatin state or transcription factor binding to reweight GWAS summary statistics, and ultimately, increase the number of loci within a high confidence interval. One example of a statistical mapping approach, part of the CARDIoGRAMplusC4D meta-analysis, used a functional GWAS (fGWAS) approach [11]. fGWAS approaches incorporate chromatin accessibility profiles [83] and compute the values as a posterior probability of association (PPA). The study identified ~20 loci believed to be primarily enriched within endothelial cells (ECs), with a strong prioritisation (PPA > 0.5). One example is rs9349379 at the *PHACTR1* locus: a gene associated with tubule formation and endothelial cell survival [84,85].

### 2.2. Expression Quantitative Trait Loci

Expression Quantitative Trait Loci (eQTL) provide an insight into the genetic component underlying gene expression and data are often examined using two publicly available databases: Genotype-Tissue Expression Project (GTEx) and Stockholm-Tartu Atherosclerosis Reverse Networks Engineering Task (STARNET). GTEx was designed from eight distinct, post-mortem human tissue samples [86] and the more recently developed STARNET provides CAD-specific gene expression associations within seven relevant tissue types [87]. Researchers utilise these and similar datasets to look at their impact on gene expression across numerous tissue types to inform their prioritisations, and colocalisation can be performed between CAD GWAS loci and eQTL signals as a method of fine-mapping risk loci.

Such an analysis found *SIPA1*, *TCF21*, *SMAD3*, *FES* and *PDGFRA* eQTLs to colocalise with CAD associations in human coronary artery smooth muscle cells [88]. Similar associations were also found with rs2820315 and *LMOD1* in smooth muscle cells (SMCs) [89] and a *BMP1* candidate variant, rs73551707, was found to be a highly significant eQTL in aortic artery tissue [90]. The latest CAD meta-analysis [11] examined eQTL signals arising from lead CAD signals using the GTEx and STARNET databases, showing a number of these present in vascular tissues, and additional data have implicated endothelial cells [42] and VSMCs specifically [91] (Table 1).

The use of statistical approaches incorporating genomic annotations and eQTL data are likely to play a large role in narrowing the lists of potential functional candidates. However, to fully characterise the effects of functional variants, laboratory tools are needed. Several recent tools that have aided the characterisation of vascular-specific CAD variants will be explored.

### 2.3. Massively Parallel Reporter Assays

Massively Parallel Reporter Assays (MPRAs) enable high throughput analysis of the effects of synthetic DNA libraries on the expression of a reporter gene, in a cell-specific manner [92]. There are several iterations of MPRA-based techniques to identify cis-regulatory elements (CREs), and most have barcoded candidate DNA libraries which are embedded within the untranslated region of a reporter gene, driving its own transcription [93,94,95]. The self-transcribing active regulatory region with sequencing (STARR-seq), a method designed to directly quantify the regulatory strength of transcriptional enhancer sequences [96], has been applied to the characterisation of functional CAD SNPs post-GWAS [97]. From a probe set designed to cover CAD GWAS variants, 14 high-confidence functional regulatory SNPs associated with CAD were reported. Amongst these associations, rs17293632, a variant located within an enhancer region for *SMAD3* in ECs was shown to alter gene expression. Additionally, a previously characterized SNP, rs17114036, which alters enhancer activity for *PLPP3*, had effects on gene expression in the STARR-seq assay. Integrating MPRA-based techniques with GWAS data has proven to be valuable in advancing our understanding of the regulatory mechanisms underlying CAD pathogenesis and deciphering the functional implications of CAD-associated SNPs. One drawback with MPRA studies, as with all reporter assays, is the lack of genomic context such as chromatin organisation. For this reason, it is often used as a large-scale prioritisation tool subsequent to further detailed studies.

### 2.4. Genome Editing

#### Knock-Out (CRISPRko)

Genome editing has become a fundamental practice for unravelling the functional implications of genomic loci. A commonly used tool is clustered regularly interspaced short palindromic repeat-associated nuclease 9 (CRISPR-Cas9). The technique functions via RNA-targeted DNA cleavage, resulting in double-stranded breaks (DBS) and when paired with a template, homology-directed repair rebuilds the DNA strand with the introduction of specific modifications [98]. In the absence of a reference template, non-homologous end joining (NHEJ) repair mechanisms take place. The error-prone NHEJ leads to the disruption, and knock-out (CRISPRko), of the targeted genomic region [99]. For the non-coding genome, this can be applied to SNPs located within, or neighbouring, CREs. For example, the consequences of rs6903956-targeted CRISPRko highlighted the relationship between the risk allele and endothelial injury via a weak promoter of *CXCL12* [100]. In a similar study, the CRISPR-mediated deletion of the enhancer overlapping a *SMAD3* intronic SNP, rs17293632, resulted in the significant reduction in *SMAD3* expression in ECs [97]. Similarly, deletion of a region containing rs7246865 resulted in a reduced expression of *HAUS8* and *MYO9B* in both ECs and loss of *MYO9B* in SMCs [11].

### 2.5. Transcriptional Modifications CRISPR

CRISPR approaches have become more advanced, which has led to intricate designs often using biologically inactivated Cas9 (dCas9) or a nickase (nCas9) that only cuts one strand of the DNA target [101,102]. The modified Cas enzymes still possess the capabilities to bind to the intended target site; however, there is no generation of DSBs. Additionally, researchers can obtain more control over the modifications to the genome, incorporating transient transcriptional modification or efficient single-base alterations [103,104].

Transcriptional modifications provide a means to dissect the non-coding genome by reversible interference (CRISPRi) or activation (CRISPRa) of CREs, [105]. By fusing the dCas9 enzyme to a repressor (most often KRAB) domain [106], downregulated gene expression can occur, which researchers have applied to numerous EC-specific studies. An investigation was conducted of two prioritized SNPs within the CAD 1p32.2 locus: rs17114036, situated within an enhancer region, and rs2184104, present at a transcription start site. rs17114036-targeted gRNAs significantly reduced expression of *PLPP3*, resulting in an increase to LPA-induced E-selectin expression and leukocyte adhesion. On the other hand, the rs2184104-targeted gRNA did not provide any notable effect [32]. In another study, the group-validated enhancer activity at three different genomic loci, rs12028528, rs7975658 and rs6825977, resulted in a reduction of *KIF26B*, *FGD6*, and *VEGFC* expression, respectively [42].

In contrast, dCas9 can be fused to activator domains (i.e., VP64) to induce an upregulation effect within the region of interest [107]. Use of CRISPRa was used as part of a pooled CRISPR screening, with single-guide RNA targeting of the rs12906125 locus resulting in the upregulation of both *FURIN*, which is associated with increased susceptibility to atherosclerotic mechanisms and *FES*, responsible for controlling cellular processes such as growth and adhesion [29].

### 2.6. Single-Base CRISPR

Base editing (BE) and prime editing (PE) are newer tools for genome editing and validating SNPs. These techniques allow researchers to directly and selectively manipulate individual nucleotides to develop a precise model for understanding their functional consequences [29,108]. In BE, investigators can systematically introduce the desired C > T or A > G transitions in the target genomic loci corresponding to the identified SNPs of interest, by employing cytosine base editors (CBEs) or adenine base editors (ABEs), respectively [109]. At the rs12906125 locus, ABE machinery introduced the homozygous (G/G) risk genotype to validate a causal gene. The resulting response to TNFα stimulation proved to be genotype-dependent for *FES*, whereas *FURIN* activity was unaffected, potentially identifying *FES* as the causal gene [29].

PE provides a more efficient and flexible process for single-base pair editing than BE by encompassing all 12 single base modifications. It requires a programmable prime editor guide RNA to drive the prime editor to its target [108]. Despite the greater potential over BE mechanisms, PE has not currently been employed in validation studies for CAD-associated variants. As a relatively new technique, PE will require a process of optimisation to become the leading tool in genome editing, and researchers are working towards overcoming current complications [110,111].

## 3. Therapeutic Potential

With ever larger GWAS meta-analyses for CAD and increasingly sophisticated tools to examine functional variants such as genome editing, attention is now directed into investigating how best to mine the findings of GWAS studies to facilitate the development of novel therapeutics. Whilst treatments for certain pro-atherogenic traits such as LDL-C can be administered relatively easily based on simple blood tests, traits related to vascular processes will inevitably be harder to identify and treat. With risk variants potentially having opposing roles related to angiogenesis or vessel morphology, it may be that polygenic risk scores could be determined related to vascular involvement for CAD. A study which looked at this possibility examined a panel of VSMCs to examine eQTLs, splicing QTLs and cell behaviour assays (including proliferation, migration and apoptosis), identifying 84 genes with eQTLS that colocalised with CAD signals [112]. These genes showed a combined polygenic effect of ~6% on VSMC behaviour. From these, 38 genes were recognised as druggable targets, including several genes in the TGFβ/BMP pathway (*TGFB1*, *SMAD3*, *BMP1* and *BMPR2*), indicating potential for vascular-based therapeutics.

The number of CAD risk genes involved with angiogenesis, including *VEGFA* and *FLT1*, shows a clear link between these pathways and disease onset or progression. The mechanisms by which pro-angiogenic factors affect disease pathology are not fully established, but it may be that atherogenesis within plaques facilitates an influx of inflammatory cells increasing the risk of plaque rupture. On the other hand, angiogenesis may help revascularisation of the ischaemic myocardium in CAD patients [113]. Therapeutics targeting angiogenic pathways have not currently been met with success for CAD-related traits. Indeed, a clinical trial that targeted VEGF using the inhibitory antibody Avastin in cancer patients noted an increased risk of thromboembolism, myocardial infarction and deep vein thrombosis [114]. It is hoped that a greater knowledge of angiogenic targets, and a personalised medicine approach to therapeutics based on an individual’s genetic background may provide future opportunities in this area.

GWAS loci have provided a number of pathways related to vascular function that could offer alternative avenues for therapeutics. One of the loci discussed, *DHX38*, plays a potential role in premature senescence in the endothelium [29] and it is believed that senescence plays an important function in atherosclerosis with senescent cells collecting at atherosclerotic blood vessels [115]. Studies in mice using senolytics have shown delayed progression of atherogenesis, indicating a potential novel drug target for the disease [116].

Whilst research proceeds into novel vascular targets for CAD, there will be a concurrent need to develop the tools to deliver such therapeutics in a targeted way. This may be facilitated by reagents such as monoclonal antibodies, antisense oligonucleotides, and CRISPR-based tools that could target RNA production. With the list of potential CAD targets increasing with each new GWAS study, and the addition of increasingly diverse populations examined, the promise of novel treatments for CAD is becoming a realistic prospect.

## 4. Conclusions

In order to identify new ways to treat or prevent CAD, we must fully understand the complex mechanisms that lead to its pathogenesis. GWAS has provided confirmation that established pathways such as lipid metabolism are key therapeutic targets, but also revealed a number of diverse mechanisms could be involved. The role of the many GWAS loci in vascular pathways is yet to be fully realised, but through the use of ever larger cohorts and distinct populations, we are beginning to identify loci in common pathways relating to specific vascular processes or vascular cell types. With advanced molecular techniques such as genome editing now becoming commonplace, and high-throughput technologies such as MPRA able to pinpoint functional variants on a large scale, the much-needed characterisation of GWAS variants is likely to increase at a faster pace. Combined with the increase in large multi-omic datasets, including transcriptomics, proteomics and single-cell data, translation of GWAS signals into novel therapeutics will soon become the new focus for CAD genomic research.

## Figures and Tables

**Table 1:** CAD GWAS genes implicated in vascular processes through eQTL analysis.

Lead Variant	GTEx		STARNET		EC ^a^	VSMC ^b^
	Artery	Gene	Artery	Gene		
rs28435150	Tibial	*FHL3*	AOR	*FHL3*		*INPP5B*
rs61776719	Tibial	*FHL3*	MAM	*FHL3*		*FHL3*
rs56170783					*PPAP2B*	
rs11585169			MAM	*ENSA*		
rs11204693	Tibial	*ARNT*	MAM	*ARNT*		*GOLPH3L*
rs12568757	Tibial	*ARNT*	MAM	*ARNT*		*CTSK*
rs10888395	Tibial	*CTSS*	MAM	*ARNT*		*CTSS*
rs1196456	Tibial	*SNX27*				*TDRKH-AS1*
rs11810571	Aorta	*TDRKH-AS1*	AOR	*ARNT*		*GOLPH3L*
rs12741323	Tibial	*NME7*	AOR	*ATP1B1*		*NME7*
rs61806987	Aorta	*NME7*				
rs1057239						*KIAA0040*
rs6700559	Tibial	*RP11-92G12.3*	AOR	*DDX59*		*DDX59-AS1*
rs17163363	Tibial	*RP11-378J18.8*				
rs16986953	Tibial	*OSR1*				
rs6736093	Tibial	*RP11-399B17.1*	AOR	*TMEM87B*		*TMEM87B*
rs148812085		*CARF*	MAM	*AC023271.1*		
rs6804986	Tibial	*ZNF589*	AOR	*ZNF589*		*NME6*
rs34759087			MAM	*SHISA5*		
rs6800032	Tibial	*PCCB*	AOR	*SLC35G2*		*NCK1-DT*
rs185244	Aorta	*MRAS*	AOR	*ESYT3*		
rs357494	Aorta	*ARHGEF26*	MAM	*ARHGEF26*		
rs4266144	Coronary	*LINC00881*	AOR	*TIPARP*		
rs781663			AOR	*REST*		*REST*
rs2127821	Tibial	*RP11-33B1.1*	AOR			*AC093752.1*
rs13124853	Tibial	*ZNF827*	MAM	*ZNF827*		
rs6841581			AOR	*EDNRA*		
rs374218	Aorta	*SNHG18*	AOR	*SEMA5A*		*SNHG18*
rs17263917	Aorta	*SEMA5A*	AOR	*CTD-2201E9.1*		
rs2910686	Tibial	*ERAP2*	AOR	*ERAP2*		*ERAP2*
rs112949822			AOR	*FER*		
rs9349379	Tibial	*PHACTR1*	MAM	*GFOD1*		
rs1034246	Aorta	*PTK7*				
rs9443626	Tibial	*IRAK1BP1*	AOR	*IRAK1BP1*		*IRAK1BP1*
rs35510806					*CENPW*	
rs2492304	Tibial	*SLC2A12*				
rs10951983	Tibial	*DAGLB*				*DAGLB*
rs1019307	Tibial	*TMEM106B*				*TMEM106B*
rs2107595	Aorta	*TWIST1*	MAM	*AC003986.6*		
rs17142613						*MACC1*
rs2215614			AOR	*TBX20*		
rs56408342	Aorta	*BMP1*	AOR	*BMP1*		
rs17566555	Tibial	*CAMK1D*				
rs9337951			MAM	*KIAA1462*		
rs55753709	Tibial	*PLCE1-AS1*				
rs884811			AOR	*LOXL4*		
rs6598075	Tibial	*RP11-326C3.16*	AOR	*RIC8A*		*RIC8A*
rs360153			MAM	*SWAP70*		
rs633185	Aorta	*ARHGAP42*	MAM	*ARHGAP42*		
rs4754694	Tibial	*TMEM133*	MAM	*ARHGAP42*		*ARHGAP42*
rs2839812	Aorta	*PDGFD*	AOR	*PDGFD*		*PDGFD*
rs1177562	Tibial	*HMBS*	MAM	*VPS11*		*AP003392.4*
rs17813323	Tibial	*YEATS4*				*YEATS4*
rs2681472	Tibial	*ATP2B1*	MAM	*ATP2B1*		
rs11107903	Aorta	*FGD6*	AOR	*FGD6*		*FGD6*
rs7133378	Tibial	*DNAH10OS*		*CCDC92*		
rs7991314	Aorta	*N4BP2L2*	MAM	*ATP8A2P2*		
rs712486	Tibial	*HAUS4*	AOR			*HAUS4*
rs10131894	Tibial	*EIF2B2*	MAM	*EIF2B2*	*EIF2B2*	*MLH3*
rs1043674	Aorta	*EIF2B2*	MAM	*EIF2B2*		*NEK9*
rs4903284	Aorta	*EIF2B2*	MAM	*EIF2B2*	*EIF2B2*	
rs36033161	Aorta	*HHIPL1*				
rs7403103	Tibial	*TRIP4*	AOR	*TRIP4*		*TRIP4*
rs56062135						*SMAD3*
rs62011052	Aorta	*ADAMTS7*	AOR	*ADAMTS7*		*ADAMTS7*
rs7173743	Aorta	*ADAMTS7*	MAM	*ADAMTS7*		
rs1807214	Aorta	*HAPLN3*	AOR	*ABHD2*		
rs8032315	Aorta	*FES*				*FES*
rs7183988	Aorta	*FURIN*		*FES*		*FES*
rs1894400	Aorta	*FES*			*FES*	
rs1800775			AOR	*AMFR*		*CETP*
rs7195958	Tibial	*DHODH*	MAM	*DHODH*		*DHODH*
rs1050362	Tibial	*DHODH*	AOR	*DHX38*		*DHX38*
rs8046696	Tibial	*BCAR1*	AOR	*BCAR1*		*CFDP1*
rs7500448	Aorta	*CDH13*	AOR	*CDH13*		
rs4790881			MAM	*SMG6*		
rs12936927	Aorta	*AC122129.1*				*TOM1L2*
rs7207292	Tibial	*EFCAB5*	AOR	*EFCAB5*		*EFCAB5*
rs2074164	Tibial	*DHX58*	MAM	*DHX58*		*DHX58*
rs4792923	Tibial	*NAGLU*	MAM	*NAGLU*		*NAGLU*
rs5820757	Tibial	*ZNF652*				
rs4794006	Tibial	*SUMO2P17*	MAM	*UBE2Z*		*ATP5MC1*
rs11079536	Tibial	*TEX2*			*PECAM1*	
rs2909217	Tibial	*PRKAR1A*				
rs11663411	Tibial	*LMAN1*				*LMAN1*
rs35562870	Tibial	*MARCH2*	MAM	*MARCH2*		*MARCH2*
rs7246865	Tibial	*MYO9B*				
rs10410487						*MAP1S*
rs2972445	Aorta	*ZNF571-AS1*	MAM	*ZFP30*		*ZFP30*
rs10409487	Tibial	*CTD-3220F14.3*	MAM	*ZFP30*		*ZFP30*
rs11466359	Tibial	*AXL*			*AXL*	
rs1800469	Tibial	*B9D2*				
rs2241709	Aorta	*EXOSC5*	MAM	*DMAC2*	*BCKDHA*	
rs8108474			AOR	*DMPK*		
rs73354869	Tibial	*LINC00189*				*MAP3K7CL*
rs28451064			AOR	*AP000318.2*		
rs35219138	Tibial	*PDXK*				*RRP1B*
rs71313931	Aorta	*ARVCF*	AOR	*ARVCF*		*ARVCF*
rs468224	Tibial	*THOC5*	AOR	*THOC5*		*THOC5*

^a^ data derived from, overlapping HAEC eQTLs with CAD-associated GWAS significant SNP data from the GWAS catalogue (as of August 2019) [42]. ^b^ data derived from 175 identified GWAS-significant loci (as of June 2020) co-localised with VSMC eQTLs [91]. Remaining variant data are derived from [11]. AOR, atherosclerotic aortic root. MAM, free internal mammary artery. Loci ordered by chromosomal position.

## Data Availability

Not applicable.

## Supplementary Materials

The following supporting information can be downloaded at: https://www.mdpi.com/article/10.3390/cells12182232/s1, Table S1: Summary of genome-wide significant CAD risk loci.
