# Supplementary material for: The Genetics of Coronary Artery Disease: A Vascular Perspective
Source: Cells. 2023 Sep 8;12(18):2232. doi: 10.3390/cells12182232 (PMC10527262; doi:10.3390/cells12182232)
Supplement: Supplementary file 1 [file cells-12-02232-s001.zip › cells-2514304-supplementary.pdf]

cells MDPI

Review

The Genetics of Coronary Artery Disease: A Vascular Perspective

Leon N. K. Quaye †, Catherine E. Dalzell †, Panos Deloukas * and Andrew J. P. Smith

William Harvey Research Institute, Faculty of Medicine and Dentistry, Queen Mary University of London, London EC1M 6BQ, UK; l.quaye@qmul.ac.uk (L.N.K.Q.); c.dalzell@smd20.qmul.ac.uk (C.E.D.); a.s.smith@qmul.ac.uk (A.J.P.S.)
* Correspondence: Correspondence: p.deloukas@qmul.ac.uk
† These authors contributed equally to this work.

Table S1. Summary of genome-wide significant CAD risk loci.

Lead Variant	Mapped gene	Chromosome	Position	Effect allele	Non-effect allele
rs2843152	*SKI*	1	2,245,570	C	G
rs7413494	*PRDM16*	1	2,985,885	C	G
rs2493298	*PRDM16*	1	3,325,912	A	C
rs12046497	*RPS6KA1, DPPA2P2*	1	26,847,640	T	C
rs79598313	*KDF1*	1	27,284,913	T	C
rs61776719	*FHL3*	1	38,461,319	A	C
rs12047439	*FOXO6, SCMH1*	1	41,809,640	A	T
rs2152314	*CCDC30*	1	42,946,462	T	C
rs6656344	*CCDC30*	1	42,948,585	A	C
rs34232196	*BSND*	1	55,489,542	T	C
rs11591147	*PCSK9*	1	55,505,647	T	G
rs472495	*PCSK9*	1	55,521,313	T	G
rs11206803	*PPAP2B*	1	56,877,509	T	C
rs56170783	*PPAP2B*	1	57,016,131	A	C
rs71646019	*LOC100131060*	1	59,433,354	T	C
rs12733512	*LINC01358, FGGY-DT*	1	59,646,978	T	C
rs12740374	*CELSR2*	1	109,817,590	T	G
rs1230666	*MAGI3*	1	114,173,410	A	G
rs61797068	*NGF*	1	115,902,514	C	G
rs67807996	*OTUD7B*	1	149,995,265	A	G
rs11585169	*MCL1*	1	150,572,037	A	T
rs12143614	*SETDB1*	1	150,932,696	A	T
rs6686750	*IL6R*	1	154,419,843	A	G
rs61806987	*NME7*	1	169,314,833	A	G
rs4650716	*KIAA0040*	1	175,128,711	A	C
rs2285219	*KIAA0040*	1	175,130,983	A	T
rs12022672	*DENND1B*	1	197,588,006	A	A
rs12048743	*DSTYK*	1	205,114,873	C	C
rs60154123	*HHAT*	1	210,468,999	T	C
rs17163363	*MIA3*	1	222,828,704	T	C
rs56236159	*RPS7, COLEC11*	2	3,636,478	T	T
rs2715879	*ASAP2*	2	9,515,551	A	G
rs16986953	*FLJ12334*	2	19,942,473	A	G
rs515135	*APOB*	2	21,286,057	T	C
rs4245791	*ABCG8*	2	44,074,431	T	C

rs76866386	*ABCG8*	2	44,075,483	T	C
rs582384	*PRKCE*	2	45,896,437	A	C
rs243071	*MIR4432*	2	60,619,028	A	G
rs4346430	*NFU1, AAK1*	2	69,668,802	A	G
rs12468870	*AAK1*	2	69,679,537	C	G
rs10176176	*PARTICL*	2	85,762,048	A	T
rs6759676	*IL1F10*	2	113,836,348	T	C
rs114192718	*SAP130*	2	128,785,663	T	C
rs4954580	*CXCR4*	2	136,986,303	T	C
rs4662330	*ARHGAP15*	2	144,186,475	T	C
rs6740731	*ZEB2*	2	145,270,592	A	G
rs10928241	*TEX41*	2	145,831,428	T	C
rs35611688	*ACVR2A*	2	148,377,860	T	C
rs10930115	*FIGN*	2	164,930,382	A	T
rs1430158	*PDE1A*	2	183,262,128	T	C
rs148812085	*NBEAL1*	2	203,877,233	T	C
rs1250247	*FN1*	2	216,299,629	C	G
rs2161967	*TNS1*	2	218,680,529	T	G
rs952227	*LOC646736*	2	227,062,080	A	G
rs4140748	*PID1*	2	230,005,505	A	G
rs283485	*GIGYF2*	2	233,645,691	A	G
rs34991912	*FGD5*	3	14,926,351	T	C
rs34759087	*LAMB2*	3	49,162,284	T	C
rs77347777	*ITIH4*	3	52,848,207	T	C
rs17843797	*UMPS*	3	124,453,022	T	G
rs34330586	*PPP2R3A*	3	135,800,409	A	T
rs185244	*MRAS*	3	138,092,889	T	C
rs7622417	*ATP1B3*	3	141,625,999	C	G
rs357494	*ARHGEF26*	3	153,937,753	A	G
rs4266144	*LOC339894*	3	156,852,592	C	G
rs16853198	*MECOM*	3	168,840,179	A	A
rs11721038	*MECOM*	3	168,849,576	T	C
rs34229028	*FNDC3B*	3	172,117,455	A	AC
rs73070813	*RPL39L*	3	186,886,595	A	T
rs12641981	*GNPDA2*	4	45,179,883	T	C
rs10938398	*PRDX4P1, THAP12P9*	4	45,186,139	A	G
rs17083333	*LNX1*	4	54,572,066	T	G
rs781663	*REST*	4	57,781,754	A	G
rs12500824	*SHROOM3*	4	77,416,627	A	G
rs10857147	*FGF5*	4	81,181,072	A	T
rs36002015	*RASGEF1B*	4	82,625,720	G	GT
rs7678555	*MAD2L1*	4	120,909,501	A	C
rs7439567	*PCDH18, LINC02172*	4	138,464,842	A	A
rs13124853	*ZNF827*	4	146,784,774	A	G
rs13120678	*EDNRA*	4	148,273,397	A	G
rs6841581	*EDNRA*	4	148,401,190	A	G
rs7440763	*MAP9*	4	156,433,520	T	G
rs3796587	*GUCY1A3*	4	156,638,073	C	G
rs869396	*PALLD*	4	169,688,000	A	C
rs17263917	*SNORD123*	5	9,552,338	A	G
rs2652682	*LOC285696*	5	17,113,657	A	T

rs1032763	*BASP1-AS1, BASP1*	5	17,118,930	T	C
rs5867305*	*SKP2*	5	36,157,262	CA	C
rs4074793	*ITGA1*	5	52,193,125	A	G
rs7719168	*ARL15*	5	53,292,390	A	A
rs62362364	*ANKRD55*	5	55,441,571	C	G
rs3936510	*MAP3K1*	5	55,860,866	T	G
rs12916	*HMGCR*	5	74,656,539	T	C
rs27660	*LNPEP*	5	96,347,775	C	C
rs288173	*FBXL17*	5	107,352,294	A	A
rs112949822	*FER*	5	108,085,190	A	G
rs256277	*EPB41L4A, NREP-AS1*	5	111,406,810	T	C
rs13169691	*DMXL1*	5	118,448,279	T	C
rs4345341	*SRFBP1*	5	121,278,751	A	T
rs73796819	*RN7SL711P, PRDM6*	5	122,420,430	A	A
rs6883598	*FBN2*	5	127,926,190	A	C
rs79968656	*SLC27A6*	5	127,998,177	A	A
rs10477741	*IRF-AS1*	5	131,795,310	T	G
rs249760	*FGF1*	5	141,915,692	T	C
rs3776307	*ARHGAP26*	5	142,494,165	A	G
rs157333	*SGCD*	5	156,117,200	C	G
rs39929	*SGCD*	5	156,123,651	A	G
rs11465228	*ADAM19*	5	157,002,695	T	C
rs72836800	*FOXC1*	6	1,617,327	T	C
rs6597292	*TXNDC5, BLOC1S5*	6	7,975,259	T	T
rs9349379	*PHACTR1*	6	12,903,957	A	G
rs6909752	*HDGFL1*	6	22,612,629	A	G
rs29266	*GABBR1*	6	29,575,279	A	A
rs9266631	*MICA*	6	31,346,898	A	G
rs62397561	*MLN, LINC01016*	6	33,784,005	T	C
rs9469899	*UHRF1BP1*	6	34,793,124	A	G
rs733701	*KCNK5*	6	39,171,862	T	C
rs1034246	*PTK7*	6	43,068,370	T	G
rs6905288	*VEGFA*	6	43,758,873	A	G
rs62405422	*TFAP2B*	6	50,796,905	T	C
rs11752218	*PRIM2*	6	57,145,562	T	C
rs79717953	*SLC17A5, CD109*	6	74,404,771	T	C
rs9361867	*FAM46A*	6	82,595,959	T	C
rs11152953	*UFL1-AS1*	6	96,894,305	T	C
rs2983896	*FHL5*	6	97,029,871	A	G
rs9400480	*TRAF3IP2–AS1*	6	111,850,597	C	G
rs35510806	*CENPW*	6	126,678,331	T	TA
rs6919211	*TARID*	6	133,999,868	C	G
rs2327426	*TCF21*	6	134,202,690	T	C
rs2492304	*SLC2A12*	6	134,378,151	A	T
rs9399136	*HBS1L*	6	135,402,339	T	C
rs17080093	*PLEKHG1*	6	150,997,440	T	C
rs6932293	*SLC22A1*	6	160,535,878	T	C
rs56195640	*SLC22A2*	6	160,720,747	A	G
rs10455872	*LPA*	6	161,010,118	A	G
rs73596816	*LPA*	6	161,017,363	A	G
rs79018195	*LPA*	6	161,024,291	T	C

rs192425087	*LPA*	6	161,032,250	T	C
rs1998043	*LPA*	6	161,097,871	A	G
rs28867400	*LPA*	6	161,104,918	A	G
rs186696265	*PLG*	6	161,111,700	T	C
rs184278183	*PLG*	6	161,177,756	T	C
rs62435159	*MAD1L1*	7	1,911,281	A	G
rs10951983	*RAC1*	7	6,446,027	A	G
rs2073533	*ETV1*	7	14,029,739	T	T
rs2107595	*HDAC9*	7	19,049,388	A	G
rs10486389	*MACC1*	7	20,300,416	A	G
rs215634	*PDE1C*	7	32,369,148	A	G
rs2215614	*TBX20*	7	35,277,093	A	C
rs2107732	*CCM2*	7	45,077,978	A	G
rs1019016	*SEMA3C*	7	80,570,562	T	G
rs917191	*EIF4EP4, SEMA3C*	7	80,570,871	C	G
rs6953441	*ZKSCAN1*	7	99,617,067	A	G
rs35146811	*CNPY4*	7	99,720,994	A	A
rs12112877	*COG5*	7	106,941,324	T	C
rs13222797	*CFTR*	7	117,100,046	T	G
rs11556924	*ZC3HC1*	7	129,663,496	T	C
rs756142636	*PARP12*	7	139,760,540	T	TCCCTGCTCTC
rs3918226	*NOS3*	7	150,690,176	T	C
rs75655731*	*LINC00599*	8	9,721,394	C	G
rs1480146	*MSR1*	8	16,129,573	A	G
rs4646249	*NAT2*	8	18,260,431	T	G
rs268	*LPL*	8	19,813,529	A	G
rs894211	*LPL*	8	19,865,747	T	C
rs66778572	*SLC18A1*	8	19,916,120	A	G
rs56408342	*BMP1*	8	22,048,490	A	G
rs1510758	*DOCK5*	8	25,061,807	A	G
rs6557894	*RNA5SP258*	8	26,086,913	T	C
rs9298506	*RP1*	8	55,437,524	A	A
rs34917849	*GEM*	8	95,278,307	C	G
rs3019173	*NDUFAF6*	8	96,020,974	T	T
rs2001846	*TRIB1*	8	126,478,450	T	C
rs1536608	*DOCK8*	9	223,613	T	G
rs504564	*LINC00583, LINC01235*	9	13,711,560	A	G
rs10961206	*C9orf146*	9	13,724,051	A	T
rs10811183	*ACER2*	9	19,436,055	A	G
rs11523031	*MTAP*	9	21,843,842	A	G
rs76959412	*CDKN2B-AS1*	9	22,011,083	C	G
rs2891168	*CDKN2B-AS1*	9	22,098,619	A	G
rs6475608	*CDKN2B-AS1*	9	22,101,702	T	C
rs4617217	*WNK2*	9	95,988,968	T	C
rs1800978	*ABCA1*	9	107,665,728	C	C
rs1967604	*KLF4*	9	110,530,324	A	G
rs7873551	*ASTN2*	9	119,245,127	C	G
rs62578408	*C5, TRAF1*	9	123,712,445	C	C
rs41312891	*C5*	9	123,726,749	G	GCAAA
rs885150	*DAB2IP*	9	124,420,173	T	C
rs651007	*ABO*	9	136,153,875	T	C

rs3935875	*GPSM1*	9	139,238,824	A	G
rs17566555	*CDC123*	10	12,275,947	A	G
rs7077962	*ARHGAP21*	10	25,054,674	T	C
rs9337951	*JCAD*	10	30,317,073	A	G
rs161231	*ZEB1*	10	31,755,920	T	C
rs75082222	*NRP1*	10	33,516,373	T	TA
rs1870634	*LOC283033*	10	44,480,811	T	G
rs2457480	*CXCL12*	10	44,740,010	A	G
rs494207	*CXCL12*	10	44,741,256	A	G
rs10740732	*BICC1*	10	60,352,888	A	G
rs17680741	*TSPAN14*	10	82,251,514	T	C
rs1051338	*LIPA*	10	91,007,360	T	G
rs1223583	*PLCE1*	10	95,756,500	A	A
rs55753709	*PLCE1*	10	96,029,170	T	C
rs1867073	*CRTAC1*	10	99,793,012	A	G
rs884811	*R3HCC1L*	10	99,923,763	C	G
rs77787671	*CNNM2*	10	104,776,205	T	C
rs2067831	*OBFC1*	10	105,643,223	C	G
rs7903146	*TCF7L2*	10	114,758,349	T	C
rs646668	*AFAP1L2*	10	116,138,034	A	G
rs2218584	*PNLIPRP2, C10orf82*	10	118,408,642	T	C
rs17101521	*LINC01153, RPL19P16*	10	122,915,553	T	T
rs2672592	*HTRA1*	10	124,230,750	T	G
rs11601507	*TRIM5*	11	5,701,074	A	C
rs56210063	*DENND2B*	11	8,789,165	C	G
rs4537761	*TMEM41B*	11	9,323,353	T	C
rs360153	*SWAP70*	11	9,762,274	T	C
rs11316597	*ARNTL*	11	13,295,751	A	AT
rs6265	*BDNF, BDNF-AS*	11	27,679,916	T	C
rs7118294	*WT1*	11	32,380,521	T	C
rs2306363	*SIPA1*	11	65,405,600	T	G
rs584961	*SERPINH1*	11	75,277,628	A	G
rs633185	*ARHGAP42*	11	100,593,538	C	G
rs1892971	*MMP13*	11	102,795,606	A	G
rs2839812	*MIR4693*	11	103,673,294	A	T
rs7117780	*CWF19L2, ASS1P13*	11	107,086,123	A	G
rs10488763	*FDX1*	11	110,244,360	A	T
rs11213945	*SIK2, LAYN*	11	111,461,003	A	G
rs11410951	*PPP2R1B*	11	111,621,399	CA	C
rs964184	*ZNF259*	11	116,648,917	C	G
rs1177562	*VPS11*	11	118,949,331	T	C
rs4938809	*ARHGEF12*	11	120,363,937	T	C
rs57812455	*GLULP3,UBASH3B*	11	122,524,967	A	A
rs10790800	*ST3GAL4*	11	126,262,638	A	G
rs11220480	*ST3GAL4*	11	126,277,570	A	A
rs72447384	*C1S*	12	7,178,440	T	TATTTA
rs10841443	*LOC100506393*	12	20,220,033	C	G
rs12820154	*PPHLN1*	12	42,783,107	A	G
rs1859440	*COL2A1, SENP1*	12	48,427,329	T	T
rs2277383	*ACVRL1*	12	52,314,388	T	G
rs75160195	*LOC400043*	12	54,521,594	T	C

rs62956461	*CNPY2*	12	56,706,178	A	AT
rs143405823	*PAN2*	12	56,723,719	T	T
rs8176893	*PAWR*	12	79,999,309	A	T
rs2681472	*ATP2B1*	12	90,008,959	A	G
rs11107903	*FGD6*	12	95,507,971	A	G
rs1558802	*MIR4496, CORO1C*	12	109,036,248	A	T
rs10774625	*ATXN2*	12	111,910,219	A	G
rs4767278	*TBX3-AS1, UBA52P7*	12	115,345,978	C	C
rs34606058	*TBX3*	12	115,353,368	T	C
rs2244608	*HNF1A*	12	121,416,988	A	G
rs7133378	*DNAH10*	12	124,409,502	A	G
rs7485656	*SCARB1*	12	125,315,647	A	G
rs7296737	*SCARB1*	12	125,336,956	T	C
rs36127550	*ZNF268*	12	133,780,309	T	G
rs12864131	*CDK8*	13	27,045,939	A	G
rs17086617	*FLT1*	13	28,962,686	T	C
rs7991314	*N4BP2L2*	13	33,126,074	T	C
rs8000794	*DOCK9*	13	99,434,810	C	G
rs11617955	*COL4A1*	13	110,818,102	A	T
rs3783113	*COL4A1*	13	110,834,746	T	C
rs11619113	*COL4A1*	13	110,918,660	C	G
rs4773141	*COL4A1*	13	110,954,353	C	G
rs9515203	*COL4A2*	13	111,049,623	T	C
rs7333991	*COL4A2*	13	111,108,436	T	C
rs4907571	*MCF2L-AS1*	13	113,618,496	T	C
rs10131894	*EIF2B2*	14	75,446,879	C	G
rs1956028	*ITPK1*	14	93,507,197	T	T
rs112635299	*SERPINA1*	14	94,838,142	T	G
rs36033161	*HHIPL1*	14	100,123,487	T	C
rs3959554	*INO80*	15	41,443,924	A	A
rs11633313	*NEDD4*	15	56,135,709	A	G
rs588136	*LIPC*	15	58,730,498	T	C
rs56062135	*SMAD3*	15	67,455,630	T	C
rs112238647	*ADAMTS7*	15	79,051,705	T	C
rs7177201	*ADAMTS7*	15	79,065,380	T	C
rs7173743	*MORF4L1*	15	79,141,784	T	C
rs2683260	*CFAP161*	15	81,385,552	T	G
rs7177107	*AKAP13*	15	86,123,364	A	G
rs1807214	*ABHD2*	15	89,565,257	A	C
rs7183988	*FES*	15	91,428,589	T	G
rs17581137	*LOC145820*	15	96,146,414	A	C
rs12691049	*MYH11*	16	15,909,513	A	C
rs10852238	*GP2*	16	20,253,374	A	T
rs111806192	*XPO6*	16	28,252,382	T	G
rs9930506	*FTO*	16	53,830,465	A	A
rs12446515	*CETP*	16	56,987,015	T	C
rs9302604	*WWP2*	16	69,576,894	A	A
rs12445401	*DHX38*	16	72,148,419	A	G
rs8046696	*CFDP1*	16	75,442,143	T	G
rs16952537	*DYNLRB2*	16	80,185,366	A	G
rs10493891	*CMIP*	16	81,510,742	T	C

rs7189462	*PLCG2*	16	81,907,867	T	G
rs7500448	*CDH13*	16	83,045,790	A	G
rs55880988	*ZFPM1*	16	88,546,253	A	G
rs4790881	*SMG6*	17	2,068,932	A	C
rs12936927	*SREBF1*	17	17,726,965	T	C
rs8077530	*TOM1L2*	17	17,853,454	T	T
rs11080107	*ANKRD13B*	17	27,938,424	T	C
rs16968377	*PIP4K2B*	17	36,942,396	T	C
rs12952244	*KAT2A*	17	40,270,081	A	G
rs8068844	*PTRF*	17	40,571,284	T	C
rs9912587	*VAT1*	17	41,172,836	A	G
rs17608766	*GOSR2*	17	45,013,271	T	C
rs4643373	*IGF2BP1*	17	47,123,423	T	C
rs5820757	*ZNF652*	17	47,417,942	G	GA
rs11655024	*BCAS3*	17	59,232,365	T	C
rs11079536	*PECAM1*	17	62,392,403	T	G
rs2909217	*WIPI1*	17	66,463,985	T	C
rs2410859	*UNC13D*	17	73,841,285	T	C
rs78532451	*TRIM65*	17	73,879,092	T	C
rs8075861	*TIMP2*	17	76,915,710	A	C
rs9951447	*CTAGE1*	18	20,009,691	T	C
rs1623003	*NPC1*	18	21,165,163	T	C
rs4327120	*RN7SKP182, RPL12P40*	18	36,532,876	A	G
rs9945890	*SMAD7*	18	46,515,916	T	G
rs12965923	*LIPG*	18	47,213,682	A	G
rs11663411	*CPLX4*	18	56,960,510	T	C
rs476828	*MC4R*	18	57,852,587	T	C
rs191615952	*PLEKHJ1*	19	2,236,097	A	A
rs12185519	*UHRF1, KDM4B*	19	4,967,739	T	C
rs116843064	*ANGPTL4*	19	8,429,323	A	G
rs55997232	*LDLR*	19	11,188,117	T	C
rs10422256	*LDLR*	19	11,216,617	A	G
rs72658867	*LDLR*	19	11,231,203	A	G
rs167479	*RGL3*	19	11,526,765	T	G
rs77420750	*CLEC4OP, CYP4F36P*	19	15,979,744	A	C
rs7246865	*MYO9B*	19	17,219,105	A	G
rs10410487	*MAP1S*	19	17,829,608	T	C
rs78030362	*ELL*	19	18,575,193	A	G
rs8102320	*ZNF100*	19	21,936,885	A	G
rs73025613	*WDR87*	19	38,334,361	T	C
rs768453105	*HNRNPUL1*	19	41,790,086	G	GTTATGGTA
rs11466359	*TGFB1*	19	41,837,615	A	G
rs1800469	*B9D2*	19	41,860,296	A	G
rs429358	*APOE*	19	45,411,941	T	C
rs7412	*APOE*	19	45,412,079	T	C
rs183657985	*EXOC3L2*	19	45,742,498	T	C
rs8108474	*RSPH6A*	19	46,301,479	T	C
rs1132274	*RRBP1*	20	17,596,155	A	C
rs6088595	*NCOA6*	20	33,358,499	A	T
rs17406518	*MMP24*	20	33,799,176	A	C
rs2207132	*MAFB*	20	39,142,516	A	G

rs6102343	*ZHX3*	20	39,924,279	A	G
rs8124182	*ZNF335*	20	44,608,901	A	G
rs2008614	*PREX1*	20	47,433,150	T	C
rs4809766	*RNF114*	20	48,559,339	T	T
rs6026739	*ZNF831*	20	57,739,469	A	T
rs2064040	*NRIP1, CYCSP42*	21	16,584,341	A	G
rs28451064	*NCRNA00310*	21	35,593,827	A	G
rs149487184	*NCRNA00310*	21	35,605,863	T	C
rs35219138	*RRP1B*	21	45,117,913	A	AT
rs4819811	*CLDN5*	22	19,609,256	A	A
rs71313931	*ARVCF*	22	19,960,184	C	G
rs12484557	*CABIN1*	22	24,555,861	A	G
rs5760309	*SPECC1L*	22	24,677,831	T	G
rs6006426	*OSM*	22	30,669,883	A	G
rs4452	*SYN3*	22	33,283,257	T	C
rs137525	*SYN3*	22	33,296,139	T	C
rs139012	*SCUBE1*	22	43,623,972	A	G
rs738408	*PNPLA3*	22	44,324,730	T	C
rs5934659	*TBL1X*	X	9,578,104	T	C
rs1410127	*OPHN1*	X	67,280,381	T	C
rs398484	*CYSLTR1, HMGN1P34*	X	77,599,469	T	C
rs2066280	*VDAC1P1, RNU6-493P*	X	80,177,630	T	A
rs2342572	*UBE2DNL, SETP4*	X	84,069,371	T	C
rs7884019	*TDGF1P3, RP11-441A11.1*	X	109,809,489	C	A
rs5929743	*MAP7D3*	X	135,318,977	G	A
rs5975828	*RBMX, RP11-308D16.4*	X	135,986,549	C	T
rs147967693	*DNASE1L1*	X	153,579,387	T	C

Positions are according to GRCh37.

*Variants that did not reach 1% FDR threshold in primary meta-analysis [11, 12].
